# Dental Caries, Dental Erosion and Periodontal Disease in Children with Inflammatory Bowel Disease

**DOI:** 10.7150/ijms.83075

**Published:** 2023-04-09

**Authors:** Eda Haznedaroglu, Esra Polat

**Affiliations:** 1 Marmara University, Faculty of Dentistry, Department of Paediatric Dentistry, Istanbul, Turkey; 2 University of Health Sciences, Sancaktepe Sehit Prof. Dr. Ilhan Varank Training and Research Hospital, Department of Pediatric Gastroenterology, Istanbul, Turkey

**Keywords:** oral health, permanent dentition, nutritional habits, oral extraintestinal manifestation, gingival index

## Abstract

**Background:** There is reportedly a higher prevalence of dental caries and periodontal disease in adults with inflammatory bowel disease (IBD) than in healthy adults. Similar data for children are lacking in the literature. We aimed to evaluate the prevalence of dental erosion, dental caries, and periodontal disease in children with IBD.

**Methods:** This was a cross-sectional comparative study. Using the established criteria of the World Health Organization, oral investigations and detailed questionnaires that covered nutritional habits were completed by the same pediatric dentist for 32 patients with IBD, aged 11 to 18 years (15.53 ± 2.00), and 32 healthy controls.

**Results:** The decayed, missing, and filled tooth index showed no significant difference between the groups (p = 0.072). The frequency of consumption of salad, lemon gum, candy and sweetened milk was significantly higher in the control group (p = 0.041, 0.012, 0.001, and 0.001, respectively) than in the IBD group. No dental erosion was observed in the IBD group. Oral mucosal history determined that 20/32 patients with IBD (62.5%) had at least one oral extraintestinal manifestation. Despite no significant differences in plaque scores between the two groups, the gingival evaluation showed a much higher mean value of gingival index scores in the IBD group than in the control group (p = 0.003).

**Conclusion:** Although the number of patients included in the study is small, we can conclude that oral extraintestinal manifestations and periodontal disease are more prevalent in paediatric patients with IBD than in healthy populations.

## Introduction

Many recent studies have explored the relationship between oral health and systemic diseases 1,2. Inflammatory bowel disease (IBD) is a recurrent immune disease of unidentified etiology that effects many people globally and impairs quality of life 3,4. The most common symptoms of IBD include abdominal pain, anemia, vomiting, and rectal bleeding 5, with the oral cavity displaying a variety of signs and symptoms of the disease. Between 48% and 80% of the paediatric IBD population reveal with oral extra-intestinal manifestations (EIMs) 4,6, which most frequently include joint disorders, skin diseases, and oral lesions such as aphthous ulcers and periodontal diseases 7.

The prevalence of IBD is rising in developed countries. In addition to its increasing general prevalence, the incidence of IBD is also rising in the paediatric population, affecting both genders 8,9. Although data are lacking for undeveloped countries, the incidence of IBD is also increasing in the recently developed countries of Asia, the Middle East, and South America, where societies have become more westernized and children are being detected at earlier ages 10,11. In Turkey, the reported incidence for ulcerative colitis is 2.6 per 100000, and for Crohn's disease, it is 1.4 per 100000 12.

The etiology of IBD is multifactorial and includes the interaction of genetic, environmental, and microbial factors with immunological reactions 13,14. Dental caries is a multifactorial disorder relating previous and current caries experience, nutrition, fluoride exposure, presence of cariogenic microorganisms, salivary status, socio-demographic impacts, food texture, high-sugar beverage consumption, and long-term oral medications with xerostomic potential. It is still known as the most prevalent chronic childhood disease in the world. A Turkish National Survey reported that 61% of children aged 12 presented with caries 15.

Dietary habits and nutritional influences are critical environmental factors in the etiology of IBD, dental erosion and caries. The Western diet is characterized by high levels of refined sugar and sugar-containing soft drinks: it has long been suspected of increasing the risk of both types of IBD which are the Crohn's disease and ulcerative colitis 16,17. According to recent studies, frequent consumption of carbonated/soft drinks is the main nutritional factor related with dental erosion, whereas frequent consumption of yoghurt is a protective factor 18. Gingivitis is related with biofilm and/or endogenous hormonal variations, medications, systemic disorders, and malnutrition 19. Plaque-associated gingivitis is the most frequent form of periodontal disorder in children, whereas periodontitis is rare 2.

IBD and periodontal disease are autoimmune-based chronic infections, with different mucosal inflammatory reactions to derestricted local microbiota in a heritable condition, leading to tissue damage. More meta-analyses have found the same conclusion 20. A large-scale national study by Lin et al. found that patients with periodontal disease had higher risk of subsequent ulcerative colitis than healthy population 21.

The prevalence of dental caries and periodontal disease is informed to be superior in adults with IBD than in healthy control subjects 22. According to a recently published review by Nijakowski et al. 23, the possibility of oral health problems in patients with IBD cannot be definitely accepted because of the possible association of other factors such as nutrition, environment, and socio-demographics 23.

To the best of our knowledge, data on the incidence of dental erosion in children with IBD are lacking; caries and periodontal disease is only one article 24. Our study aimed to assess the prevalence of dental erosion, dental caries, and the periodontal diseases in children with IBD and to compare them with the results from healthy population in a dental practice.

## Materials and Methods

### Study Population

Prior to this study, the minimum sample size was determined based on the study reported by Koutsochristou et al. as 32 with G*power 3.1.9.6 version by taking impact size 1,202, α = 0.05, power (1-β) = 0.90 at a confidence level of 95% 24.

This cross-sectional comparative study comprised 32 children (20 males, 12 females) aged 11 to 18 years (mean age:15.53 ± 2.00) with IBD in the clinical remission period: (the IBD group) who provided the inclusion criteria. The inclusion criteria were being in remission period, being in permanent dentition, not smoking, and consented the child and family to participate in the study. They were followed up in the Department of Paediatric Gastroenterology at the University of Health Sciences, Prof. Dr. Ilhan Varank Training and Research Hospital in Turkey, between June 2021 and June 2022. Thirty-two systemically healthy children (20 males, 12 females) aged 11 to 18 years (mean age: 15.25 ± 1.72) who applied to the Marmara University, Dental Faculty, Paediatric Dentistry clinic for any reason, oral examination were enrolled as the control group. Both the patients and controls came from the Anatolian side of Istanbul. Participants were excluded if they, or their families, refused contribution in this study, were at primary or mixed dentition stage, smoking, systemic disease and had orthodontic or periodontal treatment within the previous year.

All procedures performed in our study including human participants were in accordance with the Declaration of Helsinki. The study was permitted by the Ethics Committee of the University of Health Sciences, Umraniye Training and Research Hospital (Number of ethical liscense: B.10.1.TKH.4.34.H.GP.0.01/135). Written informed consent was taken from parents before participants were included in the study.

### Clinical Examination

Only participants in the IBD group were assessed by a paediatric gastroenterologist. According to the IBD guidelines, we have evaluated the patients, if they are in clinical remission with Paediatric Ulcerative Colitis Activity Index (PUCAI) scoring for ulcerative colitis and Paediatric Crohn's Disease Activity Index (PCDAI) scoring for Crohn's Disease. Therefore, these scoring systems include clinical symptoms, physical examination findings and laboratory evaluation. A total score of less than 10 for PUCAI, less than 12.5 for PCDAI indicate the period of remission.

Medical history was obtained for children in both participant groups. Anthropometric measures (weight, height) were taken for both participant groups.

### Questionnaires

According to the Oral Health Surveys: Basic Methods published by the World Health Organization (WHO) 25 and the Guideline on Caries-Risk Assessment and Management for Infants, Children, and Adolescents 26 a questionnaire containing thirty three questions was designed to elicit information for each participant regarding age, sex, height, weight, level of mothers' education, mother's age at birth, number of siblings, occupation of parents, smoking status of parents, monthly family income, detailed dietary habits, oral hygiene behaviors, mouth breathing, frequency and reason for dental visits, the year of diagnosis of IBD, types of medications used, and other factors. Questionnaire was administered and completed by dental examiner prior to intraoral examination.

### Intraoral Examination

The intraoral examination assessed oral EIMs (including all soft tissues of the mouth); dental erosion; the decayed, missing, and filled tooth (DMFT) index, gingival index system, the plaque control record index (PCR), and the community periodontal index of treatment needs (CPITN). WHO criteria were used to determine DMFT scores 16. The mean DMFT value for each patient in both groups was calculated.

Dental erosion status was defined with the basic erosive wear examination index. This index is based on the clinical features of the different grades of erosive wear (score 0, no erosion; score 1, initial loss of hard tissue; score 2, distinctive defect, hard tissue loss < 50%; and score 3, hard tissue loss ≥ 50%) 27. The gingival condition of all participants was also scored according to the gingival index system, which is according to the clinical features of the different grades of gingival inflammation (0, no inflammation; 1, mild inflammation; 2, moderate inflammation-moderate redness, edema and bleeding on pressure; 3, severe inflammation with propensity to spontaneous bleeding and ulceration) 28.

Oral hygiene status was stated using the plaque control record index to evaluate the presence or absence of marginal plaques 29. Periodontal disease was described using the CPITN after dividing the oral cavity into sextants. For individuals younger than 20 years, only six index teeth are specified. All teeth of each patient were scanned, and the highest score per sextant was noted; with the following range: 0, healthy; 1, bleeding; 2, calculus; 3, probing pocket depth ≥ 4 mm but <6 mm; and 4, probing pocket depth ≥ 6 m 30.

All intraoral examinations were performed by the same clinical experienced paediatric dentist (E.H.). Before initiation of this study, an examiner calibrated this dentist for reliability through two repeated assessments of the same 10 participants in a dental practice in 2-day intervals to decrease intra-examiner error and provide measurements within less than 1 mm of error (gold standard). The kappa scores were 0.82 and 0.88.

### Statistical Analysis

Statistical analysis was conducted using IBM SPSS Statistics for Windows Version 22.0 software (IBM Corp., Armonk, NY). Results were contemplated significant at the 5% significance level (p < 0.05).

The conformity of the numerical variables to a normal allocation was tested with the Shapiro-Wilk test. Student's t-test was used to compare normally distributed numerical variables in the two groups, the Mann-Whitney U test was used to compare non-normally distributed numerical variables between the two groups, and the interrelationships between categorical variables were tested using the chi-square test 31.

## Results

### Study Population

Sixty-four participants aged 11-18 years were comprised in this study, 32 of whom had IBD (Crohn's disease: 11; ulcerative colitis: 21); 32 served as controls.

All participants with IBD included in the study were in remission (Paediatric Ulcerative Colitis Activity Index < 10, Paediatric Crohn's Disease Activity Index < 12.5). Seven patients were treated with mesalazine, one with azathioprine, 15 with mesalazine and azathioprine, four with infliximab, four with mesalazine, azathioprine, and infliximab, and one with azathioprine and infliximab.

The demographic features of the study participants are presented in Table 1A and Table 1B. Compared to the control group, patients in the IBD group showed a significantly lower level of mother's education and a higher number of siblings (p = 0.008 and p = 0.001, respectively).

The nutritional and oral hygiene routines, mouth breathing, frequency of dental visits, and professional topical fluoride application status are presented in Table 2. With regard to nutritional habits, the frequency of consumption of salad, lemon gum, candy, and sweetened milk was significantly higher in the control group (p = 0.041, 0.012, 0.001, and 0.001, respectively) than in the IBD group.

### Dental Caries and Dental Erosion Assessment

The DMFT index scores are presented in Table 3. Although not significant, the decayed tooth scores were higher in the IBD group (p=0.072, Table 3) than in the control group. Intraoral examinations revealed that 9 of 32 children in the control group (28.1%) had erosive tooth wear, but those in the IBD group had no erosive tooth wear (Table 4).

### Oral Mucosal Lesion Assessment

Oral mucosal history determined that 20 of the 32 patients with IBD (62.5%) had at least one EIM. The most common oral lesions were aphthous ulcerations (10 patients, 31.3%), angular cheilitis (9 patients, 28.1%), and mucosal cobblestoning (6 patients, 18.8%). The children in the control group had no oral mucosal lesions on examination (Table 4).

### Periodontal Disease Assessment

Although gingivitis index scores were significantly higher (p=0.003, Table 4) in patients with IBD, there was no statistically significant difference between plaque control record scores in participants with IBD and the control group (Table 4).

More than half (53.1%) the patients in the IBD group had gingivitis with calculus (CPITN score: 2), whereas 40.6% had only gingival bleeding (CPITN score: 1), 3.1% (one patient) had probing pocket depth values between 4 and 6 mm (CPITN score: 3), and 3.1% had a healthy periodontium (p=0.001, Table 4).

## Discussion

This study evaluated the status of dental caries, dental erosion, dietary and oral hygiene habits, gingival status, and periodontal treatment needs in children with IBD. To our knowledge, this is the first study on dental erosion and second study on oral and periodontal health in children with IBD.

Various studies have reported a significantly higher incidence of dental caries in adults with IBD 32-34. Koutsochristou et al 24 studied 55 participants with IBD aged 4-18 years and found that dental caries indices were statistically higher in patients with IBD than in healthy control groups. In contrast, in this study DMFT index scores were higher in patients with IBD than in controls, but the difference was not significant (p=0.072). Unlike other studies, we investigated a number of caries risk factors together. We included only participants who were at the permanent dentition stage of development, in contrast to Koutsochristou et al 24 When primary dentition changes to permanent dentition, there is an increase in the diversity of the oral microbiota and a specific set of microorganisms living in the body develops 35. Similar to our study, study by Grössner-Shreiber et al. 36 reported no significant differences in the prevalence of caries between groups.

In our study, we determined that since all patients in the IBD group were in clinical remission, they consumed sugar and sugary food drinks very rarely to prevent disease flare-ups (as advised by the paediatric gastroenterologist). However, the frequency of consumption of sugar and sweetened milk was found to be statistically higher in our control group (p=0.001 and 0.001, respectively) than in the IBD group. Eating habits is one of the most critical risk factors for caries. It is possible that dental caries may not have been found to be significant between the groups due to patients in the IBD group having better eating habits.

We did not detect dental erosion in children in the IBD group. However, 9 out of 32 children in the control group (28.1%) had erosive tooth wear, possibly as a result of their dietary habits, i.e. consuming significantly more acidic foods.

Although the absence of statistically differences in plaque index scores between the two groups in our study, the gingival evaluation showed that the mean gingival index scores in patients with IBD was much higher than that in the control group. This is in agreement with the results of the study by Koutsochristou et al. 24.

CPITN is mainly an evaluation index for the presence or absence of periodontal pockets, calculus, and gingival bleeding 21, and it requires clinical assessment. A little over half (53.1%) the patients in the IBD group had a CPITN score of 2, 3.1% had a score of 3, and 3.1% had a healthy periodontium. Among the participants in the control group, 3.1 % had a CPITN score of 2, and none had a CPITN score of 3. Although there was no disparity in oral hygiene habits between the two groups, a higher rate of periodontal disease was observed in the IBD group. This finding is similar to those of previous studies 6,32,35,37-39. Poor oral hygiene is likely to have caused the high rate of bleeding on probing in the control group.

A recent study using the full-mouth clinical periodontal assessment in a large group of patients established a relationship between IBD severity and periodontal diseases 40. According to Kitamoto et al. periodontal disease, aggravates gut inflammation by supplying the gut with both colitogenic pathobionts and pathogenic T cells 41. Although the researchers considered variations in the host immune response, the role of periodontal microorganisms in the relationship between periodontal disease and IBD remains uncertain.

According to Shazib et al. 4, between 48% and 80% of paediatric population with IBD presented with oral extra-intestinal manifestations, which are often observed in patients with active intestinal diseases 4,42. Although all participants with IBD in this study were in clinical remission and receiving medical treatment, 62.5% of them had at least one EIM: aphthous ulcerations were the most common (31.3%), followed by angular cheilitis (28.1%) and mucosal cobblestoning (18.8%). As none of these EIMs was too large, painful, or inconvenient for eating and drinking, no treatment was required. Participants in the control group had no intraoral soft tissue lesions. The mean duration of IBD was 19 months in this study.

Oral manifestations in paediatric IBD patients may be related to a longstanding or severe deficiency of specific nutrients or to a medication-induced reaction. Deficiencies in some vitamins can cause intraoral soft tissue lesions. These deficiencies may also be related to anemia. In one study, 15% of IBD patients were found to be zinc deficient 4. The ability of zinc to modify the crystal growth pathways of calcium phosphates has been exploited to control calculus formation and reduce demineralization of the enamel 43. In our study, gingivitis, angular chelitis and dental caries in patients with IBD may also be related to deficiency of some vitamins or minerals.

The primary strength of this study is that it is the first study to investigate dental erosion in children with IBD. Additionally, we explored eight categories of risk factors for caries, all at the same time. This is also the first study conducted only on paediatric patients with permanent dentition.

A limitation of this study is that patients in the active phase of IBD were not included. Further limitations were the relatively small sample size and lack of evaluation of salivary biomarkers such as pH, cariogenic microorganisms, calcium, phosphate, and zinc.

## Conclusions

Oral EIMs and periodontal disease are more prevalent in paediatric patients with IBD than in healthy populations. Dentists should familiarize themselves with the probable oral manifestations of this disease. Regular intraoral examination may be included in the multidisciplinary management of paediatric patients with IBD, and the risk of dental caries can be reduced by practicing healthy nutritional habits and proper oral hygiene. Instead of evaluating the relationship between oral EIMs and IBD, future longitudinal large-scale studies are needed to determine whether oral EIMs are the cause or not. Preventive dentistry is needed to improve the quality of life of paediatric patients with IBD.

## Figures and Tables

**Table 1A:** Demographic characteristics of IBD patients and controls

		Patients with IBD	Controls	
		n (%)	n (%)	P
**Gender**	Male	20(62.5)	20(62.5)	1
	Female	12(37.5)	12(37.5)	
**Mother's education**	No school	1(3.1)	3(9.4)	0.008*
	Primary	22(68.8)	9(28.1)	
	Secondary	2(6.3)	6(18,8)	
	High	6(18.8)	6(18.8)	
	University	1(3.1)	8(25)	
**Does mother work?**	Yes	6(18.8)	10(31.3)	0.248
	No	26(81.3)	22(68.8)	
**Does mother smoking?**	Smoking	4(12.5)	6(18.8)	0.491
	Non-smoking	28(87.5)	26(81.3)	
**Does father smoking?**	Smoking	13(40.6)	15(46.9)	0.614
	Non-smoking	19(59,4)	17(53.1)	
**Monthly family income**	<subsistence level	8(25.0)	7(21.9)	0.915
	subsistence level	20(62.5)	20(62.5)	
	>subsistence level	4(12.5)	5(15.6)	
**Birth time**	Term	30(93.8)	31(96.9)	
	Preterm	2(6.3)	1(3.1)	

*Chi-square tests

**Table 1B:** Demographic characteristics of IBD patients and controls (continued)

	Patients with IBD (n=32)		Controls (n=32)		
	Mean ± SD	Median (%25-%75)	Mean ± SD	Median (%25-%75)	P
**Age**	15.53±2.00	16.00(14.00-1700)	15.25±1.72	15.00(14.00-17.00)	0.389ỻ
**Height (cm)**	165.25±11.33	166.00(160.00-171.00)	162.94±12.19	161.50(155.00-172.00)	0.270ỻ
**Weight (kg)**	58.03±15.19	55.50(49.00-67.00)	53.09±10.46	50.00(46.50-60.00)	0.135†
**BMI**	20.95±3.90	20.68(18.20-23.18)	19.80±1.53	19.91(18.45-20.85)	0.129†
**Time of diagnosis (months ago)**	19.00±15,13	14.00(9.00-25.00)	-	-	-
**Mother's age at birth**	27.16±8.10	25.50(20.00-35.00)	26.22±4.49	27.00(23.50-28.50)	0.925 ỻ
**Number of siblings**	3.19±1.03	3.00(3.00-4.00)	2.31±0.69	2.00(2.00-3.00)	0.001* ỻ

†Student t test ỻ Mann Whitney U test

**Table 2:** Comparison Between Children with IBD and Control Population for Dietary and Oral Hygiene Habits, Mouth Breathing, Frequency of Dental Visits and Professional Topical Fluoride Application

			Patients with IBD	Controls	
			n (%)	n (%)	P
**Salad**	Seldom/Never		6(18.8)	4(12.5)	0.041*
	Twice per week		12(37.5)	11(34.4)	
	Once per day		12(37.5)	11(34.4)	
	Twice per day		0 (0)	6(18.8)	
	More than twice per day		1(3.1)	0(0)	
	More frequent		1(3.1)	0(0)	
**Lemon gum**	Seldom/Never		26(81.3)	16(50)	0.012*
	Twice per week		5(15.6)	14(43.8)	
	Once per day		0(0)	2(6.3)	
	Twice per day		1(3.1)	0(0)	
**Candy**	Seldom/Never		29(90.6)	13(40.6)	0.001*
	Twice per week		2(6.3)	15(46.9)	
	Once per day		0(0)	3(9.4)	
	Twice per day		0(0)	1(3.1)	
	More than twice per day		1(3.1)	0(0)	
**Sweetened milk**	Seldom/Never		23(71.9)	7(21.9)	0.001*
	Twice per week		6(18.8)	12(37.5)	
	Once per day		2(6.3)	9(28.1)	
	Twice per day		1(3.1)	3(9.4)	
	More frequent		0(0)	1(3.1)	
**Orange juice**	Seldom/Never		24 (75)	15(46.9)	**0.079**
	Twice per week		6(18.8)	14(43.8)	
	Once per day		2(6.3)	2(6.3)	
	Twice per day		0(0)	1(3.1)	
**Carbonated soft drink**	Seldom/Never		18(56.3)	19(59.4)	0.113
	Twice per week		13(40.6)	10(31.3)	
	Once per day		1(3.1)	0(0)	
	Twice per day		0(0)	3(9.4)	
**Snacks**	Seldom/Never		9(28.1)	3 (9.4)	0.011*
	Twice per week		9(28.1)	7(21.9)	
	Once per day		4(12.5)	13(40.6)	
	Twice per day		7(21.9)	2(6.3)	
	More frequent		3(9.4)	7(21.9)	
**Frequency of toothbrushing**		Once per day	9(28.1)	9(28.1)	0.957
		Twice or more per day	13(40.6)	12(37.5)	
		Less than once per week	10(31,3)	11(34.4)	
**Type of toothbrush**		Electric	4(12.5)	0(0)	0.016*
		Manual	28(87.5)	32(100)	
**Toothpaste**		With F	32(100)	26(81.3)	0.003*
		Without F	0(0)	6(18.8)	
**Visit of dentist**		First visit	4(12.5)	1(3.1)	0.001*
		If necessary Regularly	28(87.5) 0(0)	23(71.9) 8(25)	
**Professional Topical Fluoride**		Yes	0(0)	8(25)	0.001*
		No	32(100)	24(75)	
**Mouth Breathing**		Yes	8(25)	9(28,1)	0.777
		No	24(75)	23(71.9)	
					

*Significant results (P < 0.05) are highlighted in bold. Chi-square tests

**Table 3:** Decayed (D), Missing (M), and Filled (F) Tooth (DMF-T) Index in Children with IBD and a Control Population

	Patients with IBD (n=32)		Controls (n=32)		
	Mean ± SD	Median (%25-%75)	Mean ± SD	Median (%25-%75)	p
**DT**	8.84±5.74	8.00(5.00-9.50)	6.13±4.62	6.00(4.00-9.00)	0.072
**MT**	0.28±0.68	0(0 -0)	0.72±1.40	0(0 -1.00)	0.281
**FT**	0.78±1.26	0(0 -1.50)	1.41±1.58	1.0(0-2.50)	0.108
**DMFT**	9.88±5.76	9.00(7.50-11.00)	8.25±5.05	8.00(5.00-11.00)	0.338

Mann Whitney U test

**Table 4:** PCR, GI, CPITN, Dental Erosion and Mucosal Lesions in Children with IBD and a Control Population

		Patients with IBD	Controls	
		n (%)	n (%)	p
**PCR**	Score 0	2(6.2)	4(12.5)	0.387
	Score 1	30(93.8)	28(87.5)	
**GI**	Score 0	2(6.3)	4(12.5)	0.003*
	Score 1	21(65.6)	28(87.5)	
	Score 2	8(25)	0(0)	
	Score 3	1(3.1)	0(0)	
**CPITN**	Score 0	1(31)	3(94)	0.001*
	Score 1	13(40.6)	28(87.5)	
	Score 2	17(53.1)	1(3.1)	
	Score 3	1(3.1)	0(0)	
**Dental Erosion**	Score 0	32(100)	23(71.9)	0.001*
	Score 1	0(0)	9(28.1)	
**Angular Chelitis**	+	9(28.1)	0(0)	0.003*
	-	23(71.9)	32(100)	
**Mucosal cobblestoning**	+	6(18.8)	0(0)	0.001*
	-	26(81.3)	32(100)	
**Aphthous Ulcerations**	+	10(31.3)	0(0)	0.001*
	-	22(68.8)	32(100)	

*Significant results (P < 0.05) are highlighted in bold. Chi-square tests
